# Nutritional and medicinal properties of Star fruit (*Averrhoa carambola*): A review

**DOI:** 10.1002/fsn3.2135

**Published:** 2021-01-23

**Authors:** Kasun Lakmal, Pamodh Yasawardene, Umesh Jayarajah, Suranjith L Seneviratne

**Affiliations:** ^1^ Department of Surgery Faculty of Medicine University of Colombo Colombo Sri Lanka

**Keywords:** *Averrhoa carambola*, benefits, medicinal properties, nutrition, review, Star fruit

## Abstract

Star fruit (*Averrhoa carambola*), a popular fruit in many parts of the world, is considered to have many beneficial nutritional and medicinal effects. However, harmful nephrotoxic and neurotoxic effects have also been described. In this review, we have discussed the reported beneficial effects of star fruit, explored the potential mechanisms for such beneficial effects, and outline factors that may affect the safe level of consumption. The beneficial effects include the following: antioxidant (mediated via L‐ascorbic acid, epicatechin, and gallic acid), hypoglycemic (mediated via high fiber levels and 2‐dodecyl‐6‐methoxycyclohexa‐2,5‐diene‐1,4‐dione), hypotensive (mediated via apigenin), hypocholesterolemic (mediated via micronized fiber), anti‐inflammatory, anti‐infective, antitumor effects, and immune‐boosting effects. The presence of chronic kidney disease, gastroenteropathies, chronic pancreatitis, dehydration, consumption on an empty stomach, and higher concentration of oxalate in fruit/juice consumed predisposes to toxicity. The level of ingestion at which the beneficial effects transition to nephrotoxicity and neurotoxicity is still to be accurately ascertained. Furthermore, the relationship between the amount of star fruit ingested and the severity of toxicity is not certain and warrants further study.

## Graphical abstract

The nutritional and medical properties of star fruit include antioxidant, hypoglycemic, hypotensive, hypocholesterolemic, anti‐inflammatory, anti‐infective, and antitumor effects. However, majority of these findings are extrapolated from in vitro or animal studies. On the other hand, star fruit ingestion has also been shown to cause nephrotoxicity and neurotoxicity. Future studies should focus on the level of ingestion and conditions at which the beneficial effects become toxic.

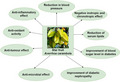

## 1 INTRODUCTION

Star fruit (*Averrhoa carambola*) is a commonly consumed fruit in both tropical and other countries. It is cultivated in many parts of the world (extensively in the South‐East Asian Region) to harvest its fruit (Khoo et al., 2010, 2016; Muthu et al., 2016). It has several nutritional and medicinal uses. Star fruit is considered a rich source of natural antioxidants and minerals (Carolino et al., 2005; Moresco et al., 2012). The star fruit may be eaten raw or be used in the preparation of juices, salads, or pickles. It is considered as a herb in several countries (Patel et al., 2015; Wang et al., 2016). As it helps with removing rust, it may be used for cleaning utensils. On the other hand, there are case reports and case series in the literature describing nephrotoxicity and neurotoxicity related to star fruit ingestion (Yasawardene et al., 2020). In this review, we have summarized the main nutritional benefits of star fruit and outlined the observed effects on different physiological processes. The beneficial pharmacological properties of star fruit and factors influencing a potential safe limit of consumption have been discussed.

## 2 MATERIALS AND METHODS

The databases PubMed, EMBASE, Scopus, LILACS, and Google Scholar were searched using the key words ‘star fruit’, OR ‘*Averrhoa carambola*’, AND ‘nutrition’, ‘medicinal properties’, ‘antioxidant’, ‘hypoglycaemic effects’, ‘hypocholesterolaemic effects’, ‘cardiovascular effects’, ‘anti‐inflammatory effects’, ‘anti‐infective effects’, ‘anti‐tumour effects’, ‘bioavailability’, ‘pharmacokinetics’ and ‘immune boosting effects’ for studies published before September 2020.

**FIGURE 1:**
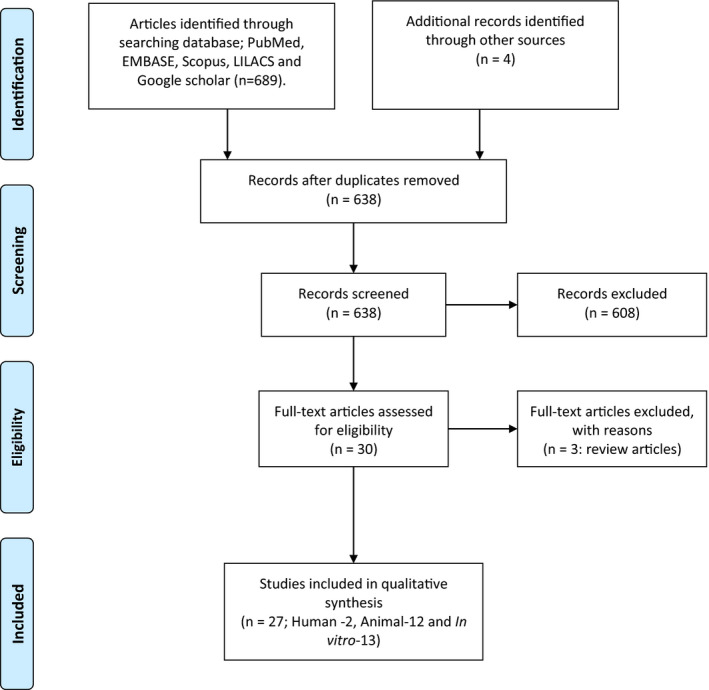
PRISMA flowchart of included studies

Results of the search strategy were examined, and studies, case reports/series that described the benefits of consuming star fruits or its juice were included in this review. Animal studies and in vitro studies focusing on the beneficial properties of star fruit were also included. Finally, a total of 2 human studies, 12 animal studies, and 13 in vitro studies were included in this review (Figure 1).

## 3 RESULTS AND DISCUSSION OF TRENDS

### 3.1 Nutritional and day‐to‐day use

There are two known varieties of *Averrhoa*: *Averrhoa carambola* and *Averrhoa bilimbi* (Ferrara, 2018). *A. carambola,* which is known as star fruit, is widely consumed in Asia. These are eaten as fresh fruit or cooked with other delicacies. They may be cooked and transformed into jams and stored in sterilized jars for long periods (Ferrara, 2018). Alcoholic beverages may be obtained by fermentation with the addition of yeasts such as *Saccharomyces cerevisiae* which release alcohol and carbon dioxide. Some communities also eat flowers and leaves of star fruit either fresh or in the cooked form (Ferrara, 2018). Star fruit contains approximately 60% of cellulose, 27% of hemicelluloses, and 13% of pectin (Khoo et al., 2010, 2016; Muthu et al., 2016). The acidity and composition of nutrients vary with the advance in maturity. According to Narain et al. (2001), the pH of the fruits become less acidic as the fruits’ ripening occurs (Narain, 2001). Furthermore, the calcium content was more at ripe stage. Factors such as titratable acidity, tannin contents, and reducing sugars varied considerably at different stages of maturity. Star fruits are a good source of vitamins and minerals. Star fruits are rich in natural antioxidants such as vitamin C, β‐carotene, and gallic acid (Khoo et al., 2010, 2016; Muthu et al., 2016). Furthermore, it is a good source of magnesium, iron, zinc, manganese, potassium, and phosphorous (Khoo et al., 2010, 2016; Muthu et al., 2016). Furthermore, it contains high amounts of fibers and low calories which may aid in controlling blood sugar (Khoo et al., 2010, 2016; Muthu et al., 2016). The second species *Averrhoa bilimbi* is native to Cuba. The fruit is small, green, and sour, and not suitable for consumption. However, it has been used as a treatment for bee sting, a stain for various dyes, antirust agent for the treatment of metals, and also as a stain remover for clothes (Ferrara, 2018). Furthermore, it is utilized as a base for various perfumes and colognes (Ferrara, 2018).

The phytochemical studies on the extracts of *Averrhoa carambola* plant leaves, fruits, and roots showed a predominant content of saponins, flavonoids, alkaloids, tannins, and pyrogallic steroids (Khoo et al., 2010, 2016; Muthu et al., 2016). Other phytochemicals such as phenols, anthocyanin and anthocyanidin, chalcones and aurones, leucoanthocyanidins, catechins, and triterpenoids were also extracted from various parts of star fruit (Silva et al., 2020).

### 3.2 Medicinal properties

**FIGURE 2:**
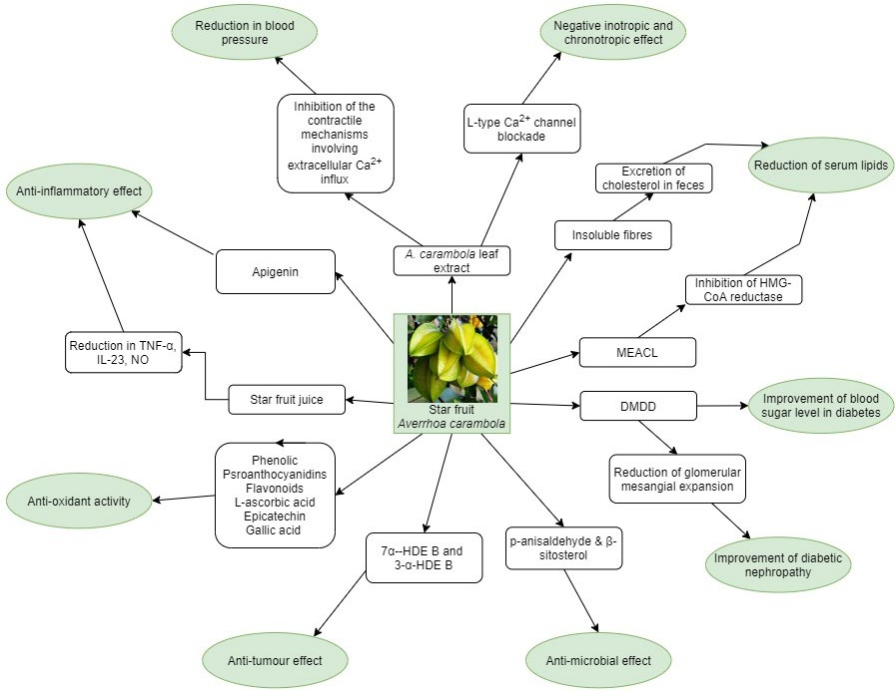
Schematic diagram of potential beneficial effects of *Averrhoa carambola* (DMDD:2‐dodecyl‐6‐methoxycyclohexa‐2,5‐diene‐1,4‐dione; MEACL: methanolic extract of *Averrhoa carambola* leaf; 7α‐HDE B: 7α‐hydroxy‐dihydro‐epideoxyarteannuin B; 3α‐HDE B: 3‐α‐hydroxy‐dihydro‐epideoxyarteannuin B, TNF‐α: tumor necrosis factor alpha, NO: nitric oxide; IL: interleukin)

Star fruits are considered to have a number of beneficial health effects. These include antioxidant, hypoglycemic, hypotensive, hypocholesterolemic, anti‐inflammatory, anti‐infective, antitumor, and immune‐boosting effects (Figure 2). Star fruits are commonly used in Ayurvedic and Traditional Chinese Medicine (TCM), and some of the clinical conditions they are used for include the following: fever, cough, diarrhea, chronic headache, inflammatory skin disorders (eczema), and fungal skin infections (Patel et al., 2015; Wang et al., 2016). The ripened fruit is also used in some countries to treat bleeding hemorrhoids.

#### 3.2.1 Hypoglycemic and antidiabetic effects

Each star fruit has a high amount of fiber, and this contributes to the beneficial effects on glucose homeostasis. The insoluble fibers inhibit the activity of α‐amylase and delays the release of glucose from starch (Chau et al., 2004). Potent hypoglycemic activity has been demonstrated in vitro. In 2007, a study performed on male Wister rats found a decrease in blood sugar level when they were fed with hydroalcoholic extract of leaves of *Averrhoa carambola*. (HELAC) (Ferreira et al., 2008). In 2016, an in vitro study on cultured pancreatic beta‐cells found the compound 2‐dodecyl‐6‐methoxycyclohexa‐2,5‐diene‐1, 4‐dione (DMDD) extracted from star fruit to attenuate inflammation and cell apoptosis. Furthermore, the same compound increased glucose‐stimulated insulin secretion (Xie et al., 2016). Using DMDD, Zheng et al. showed this compound to be effective in reducing blood sugar levels in diabetes‐induced mouse models (Zheng et al., 2013). In 2019, a study on diabetic mice found DMDD treatment to attenuate diabetic nephropathy. There was a decline in blood glucose, serum creatinine, and blood urea nitrogen levels and an increase in the quantity and density of podocytes (Lu et al., 2019). In another study from 2020, the administration of benzoquinone isolated from the roots of *Averrhoa carambola* to male Kunming mice with induced diabetes found a reduction in the blood glucose levels when compared with a control group (Qin et al., 2020). Zhang et al. showed that the beneficial effects of *Averrhoa carambola* extracts in mice with induced diabetes were probably due to inhibition of the TLR4/TGF‐β signaling pathway by active compounds like DMDD (Zhang et al., 2020). So far, the evidence lies in experimental animal studies and one in vitro study (Table 1), and thus, clinical studies are needed to assess the clinical relevant antidiabetic effects in humans.

**TABLE 1:** Summary of studies on hypoglycemic, antidiabetic, and hypocholesterolemic effects of *Averrhoa carambola*

Author (Year)	Country	Type of study	Metabolic effect	Methods	Results	Active compound identified	Possible mechanism of the effect
Human study							
Leelarungrayub, Laskin, et al. (2016), Leelarungrayub, Yankai, et al. (2016)	Thailand	Human study	Hypocholesterolemic effects	Fresh juice was prepared from each 100 g of star fruit and consumed twice daily for 4 weeks	LDL‐C level was significantly lower (*p* = .02) when compared to baseline period. However, the levels of triglyceride (*p* = .65) and cholesterol (*p* = .71) showed no difference	N/A	N/A
Animal studies							
Ferreira et al (2008)	Brazil	Animal Study (male Wistar rats)	Hypoglycemic and antidiabetic effects	Oral treatment (20 mg/kg per day) with the hydroalcoholic extract of leaves of *Averrhoa carambola*	Group receiving extract showed lower fasting blood sugar level compared to control group	N/A	Increased muscle glucose uptake
Wu et al. (2009)	Taiwan	Animal Study (hamster model)	Hypocholesterolemic effects	Micronized star fruit insoluble fiber was administered	Significant reduction in concentration of serum triglyceride (by 15.6%–17.8%) and serum total cholesterol (by 15.7%–17.0%) (*p* < .05)	Insoluble fibers	Enhanced excretion of cholesterol and bile acids in feces
Cazarolli et al (2012)	Brazil	Animal study (male Wistar rats)	Hypoglycemic and antidiabetic effects	Crude and fractional extracts of *Averrhoa carambola* leaves administered	Hypoglycemic effects in hyperglycemic normal rats.	apigenin	Stimulatory effect of apigenin on muscle glucose uptake
Zheng et al. (2013)	China	Animal study (KKAy mice)	Hypoglycemic and antidiabetic effects	Orally administrated (DMDD) (12.5, 25, 50 mg/kg BW/d) or aminoguanidine (200 mg/kg BW/d) for 8 weeks	Reduced proteinuria, serum creatinine, creatinine clearance, and serum urea nitrogen and attenuation of glomerular mesangial matrix expansion	DMDD	DMDD downregulates the NF‐kβ/TGF‐β1 pathway in diabetic glomeruli, leading to decreased ECM deposition in renal tissues
Herman‐Lara et al. (2014)	Mexico	Animal study (C57BL/6 mice)	Hypocholesterolemic effects	Micronized insoluble fiber from star fruit	Significant (*p* < .05) lowering of serum triacylglycerides, serum total cholesterol , and liver lipids	Insoluble fibers	Enhanced excretion of lipids and cholesterol in feces
Aladaileh et al (2019)	Malaysia	Animal study (high‐fat fed male Sprague Dawley rats	Hypocholesterolemic effects	Methanolic extract of *Averrhoa carambola* leaf (MEACL) was orally administered at different doses (250, 500, and 1,000 mg/kg) for five weeks.	Reduction in serum lipids in a dose‐dependent manner and healed liver tissue damage and decreased the body mass index, atherogenic index, and hepatic cholesterol and triglycerides	N/A	MEACL reduced lipid peroxidation and improved antioxidant action in the liver. HMG‐CoA reductase and lipase enzymes were suppressed.
Pham et al (2017)	China	Animal study (Kunming mice)	Hypoglycemic and antidiabetic effects	*Averrhoa carambola L*. juice was administered at 25g/kg, 50g/kg, and 100g/kg body weight once daily for 21 days	Significant reduction (*p* < .05) in fasting blood sugar level	N/A	N/A
Lu et al. (2019)	China	Animal study (mice)	Hypoglycemic and antidiabetic effects	Oral administration of DMDD (12.5, 25, 50 mg/kg body weight/d) in diabetic nephropathy induced mice	DMDD attenuated diabetic nephropathy, decreased serum creatinine and blood urea nitrogen and increased the quantity and density of podocytes	DMDD	Inhibition of inflammation and downregulates the TLR4/MyD88/NF‐κβ pathway
Qin et al., 2020	China	Animal study (male Kunming mice)	Hypoglycemic and antidiabetic effects	Administration of BACR to Streptozotocin‐induced diabetic mice (30, 60, 120 mg/kg BW) for 21 days	Reduced fasting blood glucose compared with control group, attenuation of diabetic nephropathy	Benzoquinone isolated from the roots of *Averrhoa carambola* L. (BACR)	Inhibition of the TLR4/NF‐κβ pathway
Zhang et al. (2020)	China	Animal Study (mice)	Hypoglycemic and antidiabetic effects	Four‐week treatment of DMDD at doses of 50 mg/kg, 25 mg/Kg, and 12.5 mg/kg body weight daily	Reduced fasting blood glucose, decreased urinary albumin, decreased pathological changes, and renal fibrosis	DMDD	Inhibition of the TLR4/TGF‐β signaling pathway.
In vitro studies							
Xie et al. (2016)	China	In vitro study (pancreatic beta cell line)	Hypoglycemic and antidiabetic effects	DMDD dissolved in dimethyl sulfoxide (DMSO) ‐ 10 mmol/l introduced to the cell culture	Cell viability and glucose‐stimulated insulin secretion increased in DMDD‐treated cells	DMDD	DMDD inhibited generation of inflammatory cytokines IL−6, TNF‐α, and MCP−1 Cleaved‐caspase−3, caspase−8, and caspase−9 downregulated (responsible for apoptosis)

Abbreviations: BACR, benzoquinone isolated from the roots of *Averrhoa carambola*; BW, body weight; DMDD, 2‐dodecyl‐6‐methoxycyclohexa‐2,5‐diene‐1,4‐dione; DMSO, dimethyl sulfoxide; ECM, extra cellular matrix; FF, functional food ;HFD, high‐fat diet; IFRF, insoluble fiber‐rich fraction; IL‐6, interleukin‐6; LDL, C‐low‐density lipoprotein‐cholesterol; MCP, 1‐monocyte chemoattractant protein‐1; MEACL, methanolic extract of *Averrhoa carambola* leaf; N/A, not available; NF‐κβ, nuclear factor kappa‐light‐chain‐enhance of activated B cells; TGF‐β, transforming growth factor beta; TLR4, Toll‐like receptor 4; TNF, α‐tumor necrosis factor‐α.

#### 3.2.2 Hypocholesterolemic effects

The intake of star fruit increases the removal of cholesterol and bile acids from the body. For instance, in hypercholesterolemic hamsters, the consumption of the water‐insoluble fraction of a star fruit increased the excretion of fecal total lipids, cholesterol, and bile acids (Chau et al., 2004). There was also a reduction in levels of both serum and hepatic cholesterol. A study done in 2014 by Herman‐Lara et al found that feeding of micronized star fruit fiber concentrate to C57BL/6 mice significantly lowers the concentrations of serum triacylglycerides, serum total cholesterol, and liver lipids to different extents. It did this by enhancing the excretion of lipids and cholesterol (Herman‐Lara et al., 2014). Wu et al found microsized star fruit fibers lower the concentrations of serum triglyceride (by 15.6%–17.8%) and serum total cholesterol (by 15.7%–17.0%). This effect was probably due to enhanced excretion of cholesterol (123%–126%) and bile acids (129%–133%) in feces (Wu et al., 2009). Another study found the methanolic extract of *Averrhoa carambola* leaf (MEACL) reduces the levels of serum lipids in rats fed high‐fat diets (Aladaileh et al., 2019). In addition, MEACL decreased the body mass index, atherogenic index, hepatic cholesterol, and triglycerides and increased fecal cholesterol and bile acids (Aladaileh et al., 2019). Most evidence on lipid‐lowering effects is extrapolated from animal studies (mouse models) and has so far been demonstrated in only one human study (Table 1). In 2016, Leelarungrayub studied the effect of star fruit consumption on lipid status among elderly Thai individuals. The study subjects were asked to drink 100g of fresh star fruit juice twice daily for 4 weeks. At the end of the study period, the HDL‐C level was higher (*p* = .03) and LDL‐C level was significantly lower (*p* = .02) when compared to measurements at baseline. There were no significant differences in levels of triglyceride (*p* = .65) and cholesterol (*p* = .71) (Leelarungrayub, Yankai, et al., 2016).

#### 3.2.3 Antioxidant activity

Star fruit has high antioxidant activity and is able to efficiently scavenge reactive oxygen species (ROS) and other free radicals. The fruit has high levels of flavonoids, proanthocyanidins, vitamin C, β‐carotene saponins, alkaloids, tannins, and gallic acid. It is able to inhibit the activity of cytochrome P450 3A (Hidaka et al., 2006). Several studies have analyzed its antioxidant capacity from a biochemical perspective. In 2004, Shui and Leong detected polyphenolic antioxidants in star fruit using liquid chromatography and mass spectrometry. The main antioxidant action was attributed to phenolic compounds such as L‐ascorbic acid, epicatechin, and gallic acid in gallotannin form (Shui & Leong, 2004). In another study done by the same team, the star fruit residue (following juice extraction) was found to account for around 70% of the total antioxidant activity (Shui & Leong, 2006). In a study investigating the antioxidant actions of common Mauritian exotic fruits, *A. carambola* was found to be an important source of phenolic antioxidants, and to exhibit potent health benefits in humans (Luximon‐Ramma et al., 2003). Among fruits tested from a Aizawl market of Mizoram in India, *A. carambola* was found to have moderate antioxidant activity (Ali et al., 2011). Chemical constituent analysis of *A. carambola* leaves found the antioxidant activity of the extract to be significantly correlated with its phenolic content (Moresco et al., 2012). Most of the findings so far are from in vitro studies, and only one human study (with a small sample size) has been done (Table 2). This human study assessed the effects of star fruit consumption on 27 elderly individuals. The consumption of 100g star fruit juice twice daily for 4 weeks resulted in significant improvement in antioxidant status (Leelarungrayub, Yankai, et al., 2016). There were increased total antioxidant capacity, reduced malondialdehyde and protein hydroperoxide levels (*p* < .05), and significantly increased vitamin A and C levels.

**TABLE 2:** Summary of studies on antioxidant and anti‐inflammatory effects of *Averrhoa carambola*

Author (Year)	Country	Type of study	Beneficial effect	Methods	Results	Active compound identified	Possible mechanism of the effect
Human studies							
Leelrungrayub, Laskin, et al. (2016), Leelarungrayub, Yankai, et al. (2016)	Thailand	Human Study (27 elderly individuals ‐Mean age 69.5 years)	Antioxidant effect	100 grams of fresh star fruit juice consumed daily immediately after breakfast and dinner for 4 weeks	Significant improvement in antioxidant status: increased total antioxidant capacity and reduced malondialdehyde and protein hydroperoxide levels (*p* < .05), significantly increased vitamin C and Vitamin A levels	NA	NA
Leelarungrayub, Laskin, et al. (2016)), Leelarungrayub, Yankai, et al. (2016)	Thailand	Human Study (29 elderly individuals‐Mean age 72.4 years)	Anti‐inflammatory effects	100 grams of fresh star fruit juice consumed daily immediately after breakfast and dinner for 4 weeks	Reduction in inflammatory parameters; [TNF‐α (5.92 to 1.04 pg/ml, *p* < .001), IL−23(6.23 to 0.98 pg/ml, *p* < .001), and NO (11.42 to 1.42 mmol/L, *p* < .001), but not in IL−2 (3.38 to 0.87 pg/ml, *p* = .211)	NA	NA
Animal studies							
Cabrini et al. (2011)	Brazil	Animal Study (Swiss male mice)	Anti‐inflammatory effect	Topical treatment with ethanolic extract of *A. carambola* leaves to a croton oil‐induced ear inflammation model	Reduced inflammation (edema) in a dose‐dependent manner	Apigenin	Inhibition of myeloperoxidase activity and cell migration during inflammation.
In vitro studies							
Luximon‐Ramma et al. (2003)	Mauritius	In vitro study	Antioxidant effect	Exotic fruits from Mauritius were analyzed for their antioxidant capacity, total phenolics, proanthocyanidins, flavonoids, and vitamin C content	*A. carambola* was a substantial source of phenolic antioxidants. The antioxidant activities ranged from 11 to 17 μmol/g. Total phenolic content (range) was 1,429 to 2099 μg/g fresh weight; proanthocyanidins 896 to 1,321 μg/g fresh weight; total flavonoids 103 to 148 μg/g fresh weight and vitamin C content was 144 to 190 μg/g fresh weight	Proanthocyanidins, flavonoids	NA
Shui and leong (2004)	Singapore	In vitro study	Antioxidant effect	Identify and extract compounds that contribute to total antioxidant activity in star fruit using HPLC and MS	Main antioxidants were attributed to phenolic compounds such as L‐ascorbic acid, epicatechin, and gallic acid in gallotannin form	L‐ascorbic acid, epicatechin, and gallic acid in gallotannin form	NA
Shui et al (2006)	Singapore	In vitro study	Antioxidant effect	Residue from star fruit was assessed for antioxidant activity	The residue of the star fruit accounted for around 70% of total antioxidant activity and total polyphenolic contents	NA	NA
Ali et al. (2011)	India	In vitro assessment	Antioxidant effect	Evaluated for their antioxidant activity based on the ability of the fruit extracts to scavenge 1,1‐diphenyl−2‐picrylhydrazyl‐free radicals, to reduce ferric ions determined by ferric reducing antioxidant potential assay and total phenolic content determination	The antioxidant activity was found to be 81.03 ± 1.97 g of trolox equivalent/100 g of fruit (DPPH assay), 78.770 ± 0.33 g of trolox equivalent/100 g of fruit (FRAP assay)	NA	NA
Moresco et al. (2012)	Brazil	In vitro study	Antioxidant effect	Antioxidant activity was measured using the radical scavenging assay and reducing power of iron (III) to iron (II) ions	Ethyl acetate and n‐butanol fractions showed the most antioxidant activity. Antioxidant activity exhibited a significant relationship with the phenolic content	Phenolics	NA

Abbreviations: DPPH, 2,2‐diphenyl‐1‐picryl‐hydrazyl‐hydrate; FRAP, fluorescence recovery after photobleaching; HPLC, high‐performance liquid chromatography; IL‐23, interleukin 23; MS, mass spectrometry; NA, not available; NO, nitric oxide; TNF‐α, tumor necrosis factor‐α.

#### 3.2.4 Anti‐inflammatory effects

In 2011, Cabrini et al. analyzed the potential topical anti‐inflammatory activity of star fruit leaves on male Swiss mice. Topically applied ethanolic extract of star fruit leaves reduced edema in a dose‐dependent manner in the croton oil‐induced ear edema model of inflammation. The ethanolic extract or its fractions inhibited myeloperoxidase activity, suggesting these compounds may influence cell migration during the inflammatory process (Cabrini et al., 2011). The evidence base among humans is limited and so far only one study has described the effects in humans (Table 2). In 2016, the levels of pro‐inflammatory factors were assessed in community‐dwelling elderly subjects, following the consumption of star fruit juice for 4 weeks. A reduction in tumor necrosis factor (TNF)‐alpha, interleukin (IL)‐23, and nitric oxide (NO) was observed (Leelarungrayub, et al., 2016).

#### 3.2.5 Cardiovascular effects

The C‐glycoside flavone, apigenin (also called carambola flavone), is a secondary metabolite of *Averrhoa carambola* leaves (Araho et al., 2005). Apigenin relaxes rat thoracic aorta primarily by suppressing calcium influx through both voltage‐ and receptor‐operated calcium channels (Ko et al., 1991). An aqueous extract of *Averrhoa carambola* leaves promoted a reduction in guinea pig atrial contractility and automaticity (Vasconcelos et al., 2005). This was secondary to blockade of an L‐type Ca^2+^ channel. Furthermore, it caused electrophysiological changes in the normal guinea pig heart (Vasconcelos et al., 2006). Later, Vasconcelos et al. investigated the effects of the aqueous extract of *A. carambola* leaves on cellular calcium influx by examining the left atrium of guinea pigs. The aqueous extract (1,500 μg/ml) had a positive inotropic effect (Vasconcelos et al., 2008). Soncini et al. studied the effects of the *Averrhoa carambola* aqueous extract (AEAc) of leaves, in anesthetized normotensive rats (Soncini et al., 2011). Both in vivo and in vitro, there was a lowering of arterial blood pressure. AEAc blocked extracellular Ca^2+^ influx by interacting with voltage‐operated channels, and the effects may be attributed to the presence of apigenin. At present, most of the evidence on cardiovascular effects is from in vitro and animal studies and human studies are needed to ascertain the actual clinical relevance (Table 3).

**TABLE 3:** Summary of studies on of cardiovascular effects of *Averrhoa carambola*

Author (Year)	Country	Type of study	Beneficial effect	Methods	Results	Active compound identified	Possible mechanism of the effect
In vitro studies							
Vasconcelos et al. (2005)	Brazil	In vitro (Guinea pig isolated left atrium)	Negative inotropic and chronotropic effects	Measured the atrial isometric force in stimulated left atria and determined the chronotropic changes in spontaneously beating right atria after introduction of *A. carambola* leaf extraction.	Extract abolished the contractile force in a concentration‐dependent manner and reduced the inotropic response to CaCl_2_	NA	Effect of extract on guinea pig atrial contractility and automaticity indicate an L‐type Ca2+ channel blockade.
Vasconcelos et al. (2006)	Brazil	In vitro (Guinea pig heart)	Electrophysiological effect	Aqueous extract of *Averrhoa carambola* L. leaves was used to assess electrophysiological effects	The extract induced many kinds of atrioventricular blocks (1st, 2nd, and 3rd degrees); increased the QT interval; increased the QRS complex duration; depressed the cardiac rate	NA	NA
Vasconcelos et al. (2008)	Brazil	In vitro (Guinea pig isolated left atrium	Negative inotropic effect	Aqueous leaf extract of *Averrhoa carambola* used on guinea pig left atrium in order to evaluate the inotropic effect	In the atrium, the aqueous extract (1,500 μg/ml) shifted to the right the concentration‐effect curve of the positive inotropic effect produced by L‐type calcium channel agonist	NA	Reduction of the L‐type calcium current
Soncini et al. (2011)	Brazil	In vivo and in vitro (rats and isolated rat aorta)	Hypotensive effect	This study was conducted to evaluate the hypotensive effect of the aqueous extract of *Averrhoa carambola* (AEAc) and its underlying mechanisms in the isolated rat aorta. The effect of AEAc on the mean arterial pressure (MAP) was determined in vivo in anesthetized rats	In normotensive rats, aqueous extract of *Averrhoa carambola* (12.5–50.0 mg/kg, i.v.) induced dose‐dependent hypotension	NA	Inhibition of the contractile mechanisms involving extracellular Ca2+ influx

Abbreviations: AEAc, aqueous extract of *Averrhoa carambola*; CaCL_2_, calcium chloride; i.v., intravenous; L‐type Ca^2+^, long‐lasting calcium channels; MAP, mean arterial pressure; NA, not available.

#### 3.2.6 Anti‐infective effects

In 2007, Mia et al. isolated two compounds (p‐anisaldehyde and β‐sitosterol) from the bark of *A. carambola* that strongly inhibited the growth of *Escherichia coli* and had mild inhibitory activity against fungi (Mia).

#### 3.2.7 Anti‐ulcer activity

In 2006, Goncalves et al. assessed the gastroprotective effect of a water–alcohol extract of the leaves of *A. carambola* in rats. There was significant anti‐ulcer activity in an acidified‐ethanol‐induced ulcer model. However, there was no protective activity in indomethacin and acute‐stress ulcerogenic mice models. Their overall conclusion was that *A. carambola* had low anti‐ulcer activity (Goncalves et al., 2006).

#### 3.2.8 Antitumor effects

In 2014, Singh et al. studied the prophylactic role of *Averrhoa carambola* extract against chemically induced hepatocellular carcinoma in Swiss albino mice. They found *Averrhoa carambola* extract administration led to a reduction in tumor incidence, tumor yield, and tumor burden in the hepatocellular carcinoma mouse model when compared to a control group (Singh et al., 2014). A previous in vitro study found antitumor activity of two compounds (7α‐hydroxy‐dihydro‐epideoxyarteannuin and 3‐α‐hydroxy‐dihydro‐epideoxyarteannuinon) extracted from cultured *Averrhoa carambola*, on K562 and HeLa cancer cell lines (Li et al., 2012) (Table 4).

**TABLE 4:** Summary of studies on other beneficial effects of *Averrhoa carambola*

Author (Year)	Country	Type of study	Beneficial effect	Methods	Results	Active compound identified	Possible mechanism of the effect
Animal studies							
Goncalves et al. (2006)	Brazil	Animal study (Rats)	Anti‐ulcer effect	The ethanolic extract at doses of 800 and 1,200 mg/kg per oral was given to rats with induced ulcers	Anti‐ulcer activity in the acidified‐ethanol‐induced ulcer model in rats. However, the extract did not show any activity in the indomethacin and acute‐stress ulcerogenic models. Thus, the study concluded ethanolic extract of *A. carambola* as having low anti‐ulcer activity	NA	NA
Singh et al. (2014)	India	Animal Study (Swiss Albino Mice)	Antitumor effect	Evaluate the prophylactic role of the fruit of *Averrhoa carambola* on diethylnitrosamine‐induced liver cancer in Swiss albino mice. Administration of extract was made orally at a dose of 25mg/kg body weight/day for 5 consecutive days prior to induction of the cancer	*A. carambola* extract administration resulted in a considerable reduction in tumor incidence, tumor yield, and tumor burden	NA	NA
In vitro studies							
Mia et al. (2007)	Bangladesh	In vitro study	Antimicrobial effect	Two compounds isolated from the bark of *Averrhoa carambola* were subjected to antimicrobial screening at 400 μg/disk	Inhibited the growth of *E. coli* with zone of inhibition 15 mm. In the case of fungi, mild inhibitory activity was exhibited	p‐anisaldehyde and β‐sitosterol	NA
Lie et al. (2012)	China	In vitro study	Antitumor effect	Two compounds (7α‐hydroxy‐dihydro‐epideoxyarteannuin B, and 3‐α‐hydroxy‐dihydro‐epideoxyarteannuin B) extracted from culture cells of *A. carambola* were examined for the antitumor activity on K562 and HeLa cell lines	Demonstrate that 7‐hydroxyl product exhibited stronger antitumor activity than the 3‐hydroxyl product against the K562 and HeLa cell lines	7α‐hydroxy‐dihydro‐epideoxyarteannuin B, and 3‐α‐hydroxy‐dihydro‐epideoxyarteannuin B	NA

Abbreviation: NA, not available.

#### 3.2.9 Immune‐boosting effects

Several claims have been made in the lay literature regarding possible immune function boosting effects of star fruit. At the present moment, more robust and well‐designed studies need to be carried out to explore this aspect further.

## 4 ANALYSIS CONSIDERING FUTURE RESEARCH

### 4.1 When do the beneficial pharmacological effects turn into toxic effects?

Many reports of toxicity have been described following consumption of star fruit, mainly in relation to nephrotoxicity and neurotoxicity. Several mechanisms of star fruit toxicity have been postulated; however, most evidence is based on animal studies. There has been much debate on the beneficial dose of star fruit and the dose at which it may become toxic (Yasawardene et al., 2020). Several clinical reports on star fruit toxicity suggest that the toxic dose is likely to vary depending on multiple factors such as comorbidities (chronic kidney disease, gastroenteropathies, chronic pancreatitis), levels of hydration at time of ingestion, consumption on an empty stomach, the type of star fruit consumed (sour as compared to sweet variety), and concentration of oxalate in the star fruit extract (Yasawardene et al., 2020). Caramboxin and oxalate are key structural compounds that cause toxicity. A detailed review on star fruit toxicity has described the pathophysiological mechanisms of caramboxin and oxalate in causing neurotoxicity and nephrotoxicity (Yasawardene et al., 2020). Oxalate concentrations differ between freshly prepared and commercially available juices with the pickling and diluting processes used in commercial juice production contributing to reduced oxalate concentrations when compared to freshly prepared star fruit extract (Yasawardene et al., 2020).

Furthermore, the toxic dose of star fruit in humans has not been defined. There is a wide variation in the actual amount of star fruit consumed in relation to reported toxicity. For example, from half to as many as 50 fruits at a time have been reported to cause toxic effects (Stumpf et al., 2020). In relation to volume, it ranged from 25 to 3,000 ml of undiluted start fruit juice. However, several patients have consumed the juice mixed with herbal drugs (Chen et al., 2001). Toxicity has been described in association with single consumption and long‐term ingestion. For example, consumption of 5–6 star fruits every month for 2–3 years (Abeysekera et al., 2015) and consumption of one star fruit per day for an year (Wijayaratne et al., 2018) have been reported to cause toxicity. Moreover, studies have also found that ingestion of even small amounts of star fruit on an empty stomach can lead to toxic effects (Ananna et al., 2016; Barman et al., 2016). As magnesium and calcium ions bind to oxalate in the gastrointestinal tract, their absence when on an empty stomach may lead to increased absorption of oxalate and subsequent toxicity (Azim & Salam, 2016; Chen et al., 2001). Some studies showed no association with the amount of consumed star fruit and severity of intoxication or mortality (Auxiliadora‐Martins et al., 2010; Neto et al., 2003).

Therefore, associations between the above factors and toxicity in humans need further evaluation as the toxic dose of star fruit seems multifactorial. A genetic predisposition for toxicity may also be considered in future studies. Further studies are needed to characterize the mechanisms of absorption, metabolism, and excretion of toxic molecules in star fruit (such as caramboxin and oxalate) in both healthy humans and subjects with preexisting renal impairment. Therefore, the question on the beneficial dose of star fruit and the dose at which it may become toxic remains unanswered.

It is important to note that majority of the studies describing beneficial effects of star fruit are based on experimental animal studies and in vitro animal studies. However, several claims have been made in the lay literature regarding the potential benefits of start fruit among humans. Based on the available evidence (as described in this review), there is a scarcity of human studies, and therefore, the potential benefits in humans should be interpreted with caution. Furthermore, studies on pharmacokinetics and bioavailability of star fruit are limited. Further studies in humans are required to examine the clinical relevance of these potential benefits, the dose required to reach clinically significant effects and also to assess the potential toxicity at such doses. Furthermore, until further studies are available, routine consumption of star fruit in view of achieving the above potential benefits should not be advised.

## 5 CONCLUSIONS

Star fruit extracts have demonstrated several potential beneficial medicinal properties including antioxidant, hypoglycemic, hypocholesterolemic, anti‐inflammatory, cardiovascular, antitumor, and immune‐boosting effects both in vitro and in vivo studies. However, majority of these findings are extrapolated from in vitro or animal studies. On the other hand, star fruit ingestion has also been shown to cause nephrotoxicity and neurotoxicity. At the present time, the level of ingestion at which the beneficial effects become toxic has not yet been precisely defined. The presence of chronic kidney disease, gastroenteropathies, chronic pancreatitis, dehydration, consumption on an empty stomach, and higher concentration of oxalate in the fruit/juice consumed predisposes to toxicity. Therefore, considering the lack of human studies and the reported toxicity profile, further studies in humans are necessary, specifically related to pharmacokinetics and bioavailability.

## CONFLICT OF INTEREST

None declared.

## AUTHORS’ CONTRIBUTIONS

KL, PY, UJ, and SLS designed the study. The search was conducted by KL and PY. Article titles, abstracts, and full texts were assessed by PY, UJ and KL. PY, UJ, and KL wrote the first version of the manuscript, and SLS performed the final editing. All the authors reviewed and approved the final version. All authors had full access to the data in the study and final responsibility for the decision to submit for publication.

## ETHICAL APPROVAL

Not applicable.

## Data Availability

Data sharing not applicable to this article as no datasets were generated or analyzed during the current study.
